# Redefining precision radiotherapy through liquid biopsy

**DOI:** 10.1038/s41416-023-02398-5

**Published:** 2023-08-19

**Authors:** D. B. McLaren, T. J. Aitman

**Affiliations:** 1 Edinburgh Cancer Centre, Western General Hospital, University of Edinburgh, Crewe Road South, Edinburgh, EH4 2XU UK; 2 Centre for Genomic and Experimental Medicine, Institute of Genetics and Cancer, University of Edinburgh, Crewe Road South, Edinburgh, EH4 2XU UK

**Keywords:** Tumour biomarkers, Cancer genomics

## Abstract

Precision radiotherapy refers to the ability to deliver radiation doses with sub-millimetre accuracy. It does not however consider individual variation in tumour or normal tissue response, failing to maximise tumour control and minimise toxicity. Combining precise delivery with personalised dosing, through analysis of cell-free DNA, would redefine precision in radiotherapy.

## Introduction

Around 50% of cancer patients require radiotherapy at some point in their treatment. The probability of treatment success is dependent upon the dose delivered and the relative radio-sensitivity of the tumour. The maximum dose delivered is calculated to ensure that no more than 10% of patients suffer life-changing late radiotherapy toxicity with an additional 20% reporting toxicity that still has some impact on quality of life. The current “one dose fits all” approach to radiotherapy treatment contrasts with the ethos of precision oncology in other branches of cancer medicine, which increasingly harness the power of genetic and genomic analyses to stratify patient care, monitor tumour response and detect disease progression.

Liquid biopsy - the analysis of cell-free DNA (cfDNA) and other cellular components released from dying or damaged cells into the circulation or other bodily fluids - has utility because the cellular components carry the characteristics of the tissues from which they are derived. Circulating tumour-derived DNA (ctDNA) retains the genetic and epigenetic hallmarks of the originating tumour, including somatic mutation and DNA methylation. Attractive because it is minimally invasive and repeatable, liquid biopsy is gaining traction in early detection and diagnosis, patient stratification, detection of minimal residual disease and prediction of recurrence after primary treatment [1–5].

Analysis of cfDNA has been little applied to management of radiotherapy, especially dosing or scheduling. However, the ability to quantify ctDNA and to define cfDNA tissue-of-origin in real time have the potential both to permit assessment of tumour response to radiation during a course of radiotherapy treatment and, at the same time, to measure the extent of damage to healthy tissues surrounding the targeted tumour. These advances, technically possible, would allow truly personalised adjustment to radiotherapy dose per fraction or number of fractions, based on how the patient is responding to their treatment and would transform how radiotherapy is currently delivered.

Here, we review recent data that define the mechanism and timing of cfDNA release arising from tissue damage and cell death after exposure to ionising radiation and the extent to which this can be quantified and ascribed to tissue-of-origin. Finally, we consider the work still required to validate and clinically translate these observations to new precision in radiation therapy.

## cfDNA release after exposure to ionising radiation

Cellular DNA is released into the circulation primarily from dividing, damaged or dying cells undergoing apoptosis or necrosis. In radiotherapy, ionising radiation causes direct and indirect DNA damage predominantly through single and double strand breaks [6]. If this damage cannot be corrected by cellular DNA repair mechanisms, cell death will occur by an interplay of apoptosis, necrosis, or mitotic catastrophe [7].

In one of the first studies investigating the kinetics of ctDNA release during radiotherapy, Lo et al. quantified Epstein-Barr virus (EBV) ctDNA in patients undergoing radiotherapy for nasopharyngeal cancer. Having observed that 2 out of 10 patients had a transient rise in circulating EBV DNA one week after initiation of radiotherapy, a further 5 patients were recruited, in whom EBV DNA levels were measured daily during the first week. All 5 patients exhibited a transient rise in EBV DNA in the first week of radiotherapy, followed by a fall [8]. More recently, in pre-clinical studies, Rostami et al measured ctDNA release following chemical induction of apoptosis and exposure to radiation in tumour cell lines and tumour xenograft mouse models. While there was little immediate ctDNA release after irradiation, a subset of irradiated cell lines demonstrated a rise in ctDNA after 72–96 h. Supporting this timeline, three of five irradiated xenograft mouse models also showed a delayed increase in ctDNA release between 96 and 144 h after irradiation. These findings led them to propose a model of cell death and DNA release whereby, in certain cell types, mitotic catastrophe predominates in the early response to irradiation exposure, with a peak in apoptosis and cfDNA release later at around 3–6 days following irradiation [7]. Similarly, Muhanna et al, studying a model of buccal cancer in rabbits, found an initial rise in ctDNA in the 1–3 days following initiation of radiation therapy followed by a consistent fall [9].

These studies suggest a pattern of release of ctDNA from tumour cells, in which damaged cells undergo a combination of immediate and delayed cell death, with an initial rise of ctDNA release into the circulation following exposure to radiation, and detectable ctDNA concentrations that subsequently fall as tumour bulk decreases.

## Detection, characterisation and analysis of cfDNA

ctDNA in cancer patients usually constitutes a small, frequently tiny proportion of the total circulating cfDNA. In early-stage cancer, ctDNA generally accounts for less than 1% of total cfDNA and only in late-stage cancer and in a subset of high-secreting tumours, does the proportion of ctDNA rise above 1% [10–12]. Methods of ctDNA detection must therefore be sensitive and specific to be clinically useful.

Tumour-derived somatic mutation in cfDNA can be detected by targeted or genome-wide sequencing and by multiplex and allele-specific PCR. A range of methods have been developed to maximise sensitivity and specificity including barcoding, amplification, error correction and deep sequencing [11–13]. Such methods are necessary because of the low total concentration of cfDNA in the circulation, the often low fraction of cfDNA that is tumour-derived and the molecular heterogeneity across cancer types and of individual tumours. These methods have already been successfully deployed across a range of clinical cancer studies for treatment stratification, assessment of minimal residual disease and detection of disease progression following primary treatment [1–5, 14–17]. In addition, because each tissue in the body, including tumour tissues, carries its own unique DNA methylation profile and these tissue-specific methylation profiles are retained after release of cellular DNA into the circulation, DNA methylation analysis is now recognised as a powerful tool for determining the tissue origin(s) of DNA samples including tumour-derived cfDNA [18–20].

In virus-associated cancers, detection of viral cfDNA by droplet digital PCR (ddPCR) or sequencing has been used to screen for virus-associated tumours in healthy populations and for characterisation of viral status at diagnosis. In longitudinally collected samples post-treatment, cfDNA viral titre has been successfully used as a prognostic marker and early marker of disease progression. Such approaches have shown particular value in EBV-associated nasopharyngeal cancer and human papillomavirus (HPV)-associated oropharyngeal cancers [21–24].

## Personalising radiotherapy

**Fig. 1:**
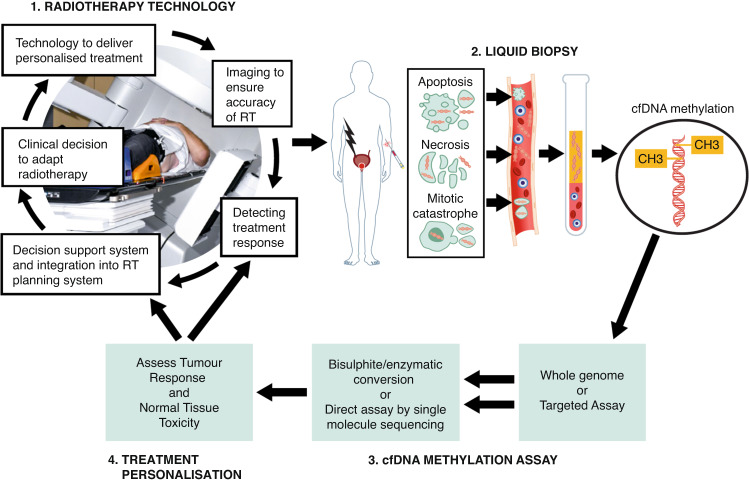
Integration of liquid biopsy into a personalised, adaptive radiotherapy workflow. For a prostate cancer patient receiving radiation treatment, shown here as an exemplar, high dose radiation is targeted at the prostate tumour with potential for toxicity to surrounding tissues. Individual cfDNA methylation results feed back on radiation dose and schedule, to maximise tumour response and minimise toxicity to surrounding tissues. RT radiation treatment.

Advances in radiotherapy technology have improved the accuracy of radiation delivery to the tumour and clinical trials have refined dosing schedules with resultant improvements in outcomes, as reviewed elsewhere [6, 25, 26]. However, toxicity remains common, in part because of the absence of biomarkers of toxicity, such that once dosing has started, it continues till the end of the schedule unless severe acute symptomatic toxicity occurs. Further, despite known variation in inter-individual and tumour radiosensitivity, manifested at the germline and gene expression level [27–29], genetic methodologies have to date impacted little on cure rates or toxicity [26]. Recent advances in understanding the mechanisms and kinetics of ctDNA release from tumours and in the ability to define cfDNA tissue-of-origin suggest that liquid biopsy has the potential to change this (Fig. 1).

In EBV-associated nasopharyngeal cancer, Lo et al. defined the kinetics of ctDNA release through measurement of EBV cfDNA [8], leading to the proposal of several groups that measurement of EBV viral load at the midpoint of radiotherapy treatment could be used as a basis for intensification or de-intensification of treatment, respectively, in those with an adverse or favourable cfDNA response [21, 23]. Similarly, Chera et al demonstrated that a favourable HPV clearance profile during chemoradiotherapy, defined as >95% clearance of circulating HPV cfDNA from a high HPV baseline copy number, was predictive of disease control in HPV-associated oropharyngeal cancer and could be the basis of trials to investigate de-intensification based on HPV clearance rate [22]. In non-virus associated cancers a number of recently published studies have demonstrated that the persistence of tumour-derived cfDNA following primary therapy, can predict those patients most likely to benefit from adjuvant therapy or subsequently relapse with early metastatic disease [4, 5, 30–33]. These results, some of which are primed for use in the clinic [3, 34], give optimism that the results in virus-associated cancers may also be applicable in non-virus associated cancers.

Of perhaps even greater novelty and applicability for radiotherapy would be the development of biomarkers of radiation-induced damage to tissues surrounding the targeted tumour. In the past five years, it has become clear that DNA methylation analysis has this capability. Extensive methylation atlases of healthy tissues and tumour types have been developed with the power to define the tissue composition of cfDNA samples and the ability to detect with high sensitivity the presence of DNA from a wide range of tissues and tumours, with demonstrated or potential application in the context of autoimmune disease, transplant rejection and early stage cancer [2, 18–20]. Showing promise for detecting damage to tissues surrounding the targeted tumour, methylation analysis has recently been reported to detect liver-derived cfDNA in patients undergoing radiotherapy treatment for right-sided but not left-sided breast cancer [35].

**Fig. 2:**
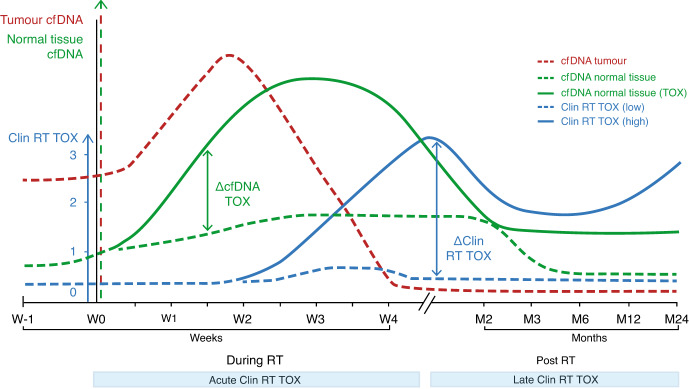
Dynamics of cfDNA release in radiotherapy. The release of tumour and normal tissue cfDNA occurs earlier than the onset of clinically apparent radiation toxicity, giving a window of opportunity for adapting radiation dose if excessive normal tissue-derived cfDNA is detected early in treatment (solid compared to dashed green line), measured as ΔcfDNA TOX. Without adaptation, the higher level of acute clinical radiation toxicity (end of week 4, solid blue line) may not be repaired, leading to subsequent life-changing permanent late radiation damage (solid blue line) by month 24. Assessment of tumour cfDNA release (dashed red line) can ensure that any reduction in normal tissue dose does not adversely impact tumour control or could possibly safely escalate the dose to tumour. Monitoring cfDNA release from tumour and normal tissue creates the ability to personalise dosage early in treatment and would redefine precision radiotherapy.

These proof-of-concept studies offer the prospect of direct tests both for tumour cell death and off-target tissue damage during radiotherapy treatments, as has also been noted in other recent reviews [10, 36]. Such tests could serve as biomarkers for adaptive radiotherapy regimes that guide treatment intensification or de-intensification based on the kinetics of clearance of circulating ctDNA and the presence or absence of cfDNA from healthy tissues surrounding the targeted tumour. However, the variable kinetics of cfDNA release in different cancer types and the currently incomplete knowledge of how different clinical radiotherapy fractionation protocols may impact on cfDNA release indicate that further study will be required to define the optimum timing of cfDNA assessment to impact effectively on radiotherapy outcomes (Fig. 2).

## Validation and clinical translation

While evidence for the value of ctDNA analysis following primary treatment with surgery, chemotherapy and radiotherapy is accumulating rapidly, the use of cfDNA analysis to guide radiotherapy dosing and scheduling during treatment is at an early stage. The existing proof-of-concept studies need confirmation in further clinical studies for example on the kinetics of cfDNA release in different tumour types and with different radiotherapy protocols. Technical advances are also required, such as development of protocols to measure DNA methylation directly in real-time, as may now be achievable by single molecule sequencing [37–39]. However, given the data from pre-clinical models and virally-induced cancers which indicate that radiation damage to tissues leads to release of detectable cell-free DNA into the surrounding milieu, it is plausible that current liquid biopsy methods may be sufficiently sensitive to provide clinically useful biomarkers of real-time tumour response and damage to healthy tissues in radiotherapy management. The confirmation of these data, being sought in several labs worldwide, would allow intra-treatment analysis of cfDNA for personalised adaptative radiotherapy scheduling, enabling more effective tumour cell death while minimising healthy tissue toxicity. Such an advance would be transformative for precision management of radiation treatments for cancer.
